# Mapping human fatalities from megafauna to inform coexistence strategies

**DOI:** 10.1038/s41598-025-04934-0

**Published:** 2025-09-30

**Authors:** Blessing Kavhu, Courage Mutema, Kudzai Shaun Mpakairi, Edson Gandiwa, Justice Muvengwi

**Affiliations:** 1 Environmental Studies, University of California, 1156 High 5th, Santa Cruz, CA 95064 USA; 2 Scientific Services Unit, Head Office, Zimbabwe Parks and Wildlife Management Authority, , Corner Borrowdale/ Sandringham Drive, Alexandra Park, Harare, Zimbabwe; 3 Institute for Water Studies, Faculty of Natural Sciences, University of the Western Cape, Cape Town, South Africa; 4 School of Wildlife Conservation, African Leadership University, Kigali, Rwanda; 5 School of Animal, Plant and Environmental Sciences, University of the Witwatersrand, Private Bag 3, Johannesburg, 2050 South Africa; 6 Department of Ecology and Evolutionary Biology, University of California Santa Cruz, California Santa Cruz, USA; 7 Department of Geography and Environmental Studies, Stellenbosch University, Western Cape Stellenbosch, South Africa

**Keywords:** Retaliatory killing, Psychosocial, Mental health, Deaths, Gettis Ord G*, Coexistence, Biodiversity, Environmental impact, Psychology and behaviour, Sustainability, Conservation biology

## Abstract

Human fatalities from human–wildlife conflict (HWC) represent a critical dimension of conservation, often triggering retaliatory actions and post-traumatic stress in affected communities. However, most studies focus on the economic implications of HWC, neglecting human fatalities which may have far-reaching long-term implications. This study investigates the spatial and temporal patterns of human fatalities caused by megafaunal species in Zimbabwe, using data collected from 2016 to 2022. Through spatial and statistical analyses based on the Getis-Ord Gi* hotspot analysis and Mann–Kendall trend test, we assess fatalities caused by six megafaunal species: Nile crocodile (*Crocodylus niloticus*), African elephant (*Loxodonta africana*), hippopotamus (*Hippopotamus amphibius*), African buffalo (*Syncerus caffer*), African lion (*Panthera leo*) and spotted hyena (*Crocuta crocuta*). The results of the study showed that crocodiles and elephants account for over 80% of human fatalities in Zimbabwe. These fatalities also significantly increased over the study period (*p* < 0.03). In contrast, fatalities involving lions, hyenas, hippos, and buffaloes showed no significant increase, indicating more stable but still concerning risks. Fatality hotspots were concentrated in Kariba, Binga and Hwange districts in northern and western Zimbabwe, highlighting areas needing urgent interventions. These insights have broader implications for HWC management across Africa, where megafaunal species frequently interact with human populations. By adopting data-driven, species-specific strategies, other countries facing similar conflicts can foster human–wildlife coexistence and improve conservation outcomes.

**Supplementary Information:**

The online version contains supplementary material available at 10.1038/s41598-025-04934-0.

## Introduction

Human–wildlife interactions occur along a continuum that includes both conflict and coexistence. While human–wildlife conflict (HWC) has historically received more attention due to its immediate socio-economic and conservation implications, there is a growing recognition of the need to frame these interactions within a broader coexistence paradigm^1,2^. Human–wildlife coexistence refers to the dynamic state where humans and wildlife share landscapes in ways that allow for the persistence of both, despite potential risks and trade-offs^1^. This approach acknowledges that not all interactions are negative and that fostering tolerance, promoting shared benefits, and integrating local values and knowledge systems can support long-term conservation goals^3^.

However, HWC poses significant challenges globally, leading to substantial biodiversity loss and severe impacts on human lives and livelihoods^4^. HWC arises when wildlife needs clash with human activities, resulting in crop destruction, livestock predation, property damage, and, most critically, human injuries and fatalities^5^. The problem is exacerbated by the increasing human population, habitat fragmentation, and the expansion of agricultural lands into wildlife habitats, making HWC a substantial challenge for wildlife conservation and human well-being^6^. In Mozambique, approximately 300 people die annually from crocodile attacks^7^. Ineffective management of HWC can lead to retaliatory killings of wildlife, particularly in response to human fatalities, undermining efforts to promote coexistence^8^. Such retaliatory actions are often fueled by the trauma, anger and resentment of vicarious victims of HWC, highlighting the importance of strategies that address post-traumatic disorders from HWC to foster coexistence^9,10^.

The nature and intensity of HWC-related fatalities vary across regions, influenced by the species involved, cultural attitudes towards wildlife, and the socio-economic conditions of human communities^11–15^. In Asia, elephants (*Elephas maximus*) and tigers (*Panthera tigris*) are frequently implicated in deadly encounters with humans^16^. Human fatalities from elephants often occur during raids on human agricultural fields^17^. Although less frequent, tiger attacks occur in areas where human settlements encroach on tiger habitats^18^. In Africa, HWC is similarly widespread, with megafauna such as African elephants (*Loxodonta africana*), lions (*Panthera leo*), hippopotamus (*Hippopotamus amphibius*), and Nile crocodiles (*Crocodylus niloticus*) frequently involved in fatal interactions with humans^19–22^. African elephants, in particular, are known to attack humans, especially in areas with high elephant densities^23^. Cultural factors often complicate these threats, as traditional practices and beliefs influence how communities perceive and respond to wildlife^3,24,25^. These conflicts have led to several mitigation strategies, including the construction of physical barriers, relocation or elimination of problematic animals, and community-based conservation programs to reduce human–wildlife encounters^26,27^. However, these measures only provide temporary relief, as wildlife may adapt, leading to more fatalities.

Zimbabwe’s extensive network of protected areas, including national parks, conservancies and safari areas, continue to support a diverse array of megafaunal species, which are often implicated in HWC^27–30^. The intensity of HWC in Zimbabwe is driven by changing land use patterns, wildlife population densities, and the socio-economic conditions of local communities^31–33^. While human populations living adjacent to protected areas frequently experience conflicts with wildlife such as crop destruction, livestock predation, and, in some cases, human fatalities^26^, it is important to recognize that these interactions are not solely negative. The presence of iconic megafauna like elephants, lions, and buffaloes also offers significant socio-economic benefits, particularly through wildlife-based tourism, which contributes to rural livelihoods and national economies^34–36^. Additionally, emerging market-based conservation mechanisms, such as biodiversity credits and payments for ecosystem services, hold promise for incentivizing coexistence and habitat protection^37^. Beyond economic gains, many African communities ascribe deep cultural, spiritual, and symbolic value to wildlife species, which fosters stewardship and positive perceptions of wildlife^37,38^.

Where human death results from HWC, it often leads to deep emotional trauma to affected families and communities^18,39^. Despite the devastating nature of such losses, the provision of psychosocial support to victims remains largely neglected^18,39^. Studies have shown that exposure to traumatic events, particularly the loss of loved ones, can lead to severe psychological distress, including post-traumatic stress disorder (PTSD), depression, and anxiety^39–41^. However, mental health intervention in the context of HWC-related human fatalities has rarely been prioritized, especially in Zimbabwe, where mental health services have historically been underfunded and underdeveloped^42,43^. As awareness of mental health issues begins to gain traction across the continent, establishing a baseline for mental health interventions, such as mapping hotspots of human fatalities due to HWC, becomes increasingly crucial. This fosters the crafting of structured psychosocial support in regions where human loss due to HWC is prevalent, and it provides guidance for targeted mental health interventions tailored to these communities.

Community-based natural resource management (CBNRM) programs have made considerable efforts to involve local communities in wildlife conservation and conflict mitigation^44–47^. However, in other areas, CBNRM programs are not fully implemented, and human settlements continue to expand into wildlife habitats, increasing the frequency of human–wildlife encounters and, consequently, human fatalities. While literature indicates the involvement of megafaunal species such as elephants, lions, buffaloes, hippos, and crocodiles in human fatalities in Zimbabwe^48–50^, no studies have explicitly quantified their contribution to human fatalities. Previous studies have mostly focused on the economic losses caused by megafaunal species through crop and livestock raids^31,51,52^. Unlike economic losses, which can be financially compensated, human life cannot be restored. This often results in greater resentment and anger among affected communities, leading to retaliatory killing of wildlife. This study seeks to address this knowledge gap by focusing on the spatial and temporal patterns of fatalities to guide strategies for human–wildlife coexistence. Understanding the dynamics of human fatalities in HWC in Zimbabwe is urgently needed to establish the circumstances under which they occur and to develop targeted measures that ensure providing psychosocial support to affected communities and public safety, reduce retaliatory killing of wildlife and facilitate coexistence. This understanding could also enhance the effectiveness of CBNRM programs by integrating data-driven approaches for problem animal control.

This study aims to contribute to the growing literature on HWC by focusing on human fatalities caused by megafaunal species in Zimbabwe. By analyzing incident records and data on human–wildlife interactions, this research seeks to identify the deadliest species and the spatio-temporal trends of these lethal encounters. The findings will provide valuable insights into species-specific risks and inform the development of effective mitigation strategies to enhance wildlife ownership in communities living in wildlife habitats globally. In addition, this study seeks to identify areas in need for psychosocial support for victims of human loss due to HWC. As mental health issues begin to gather momentum in African countries, the research sets a baseline for mental health interventions by mapping hotspots of human fatalities. By recognizing both the physical and emotional toll of HWC, this work serves as a critical step toward holistic approaches that prioritize both conservation efforts and the well-being of affected communities.

## Materials and methods

### Study area

**Fig. 1:**
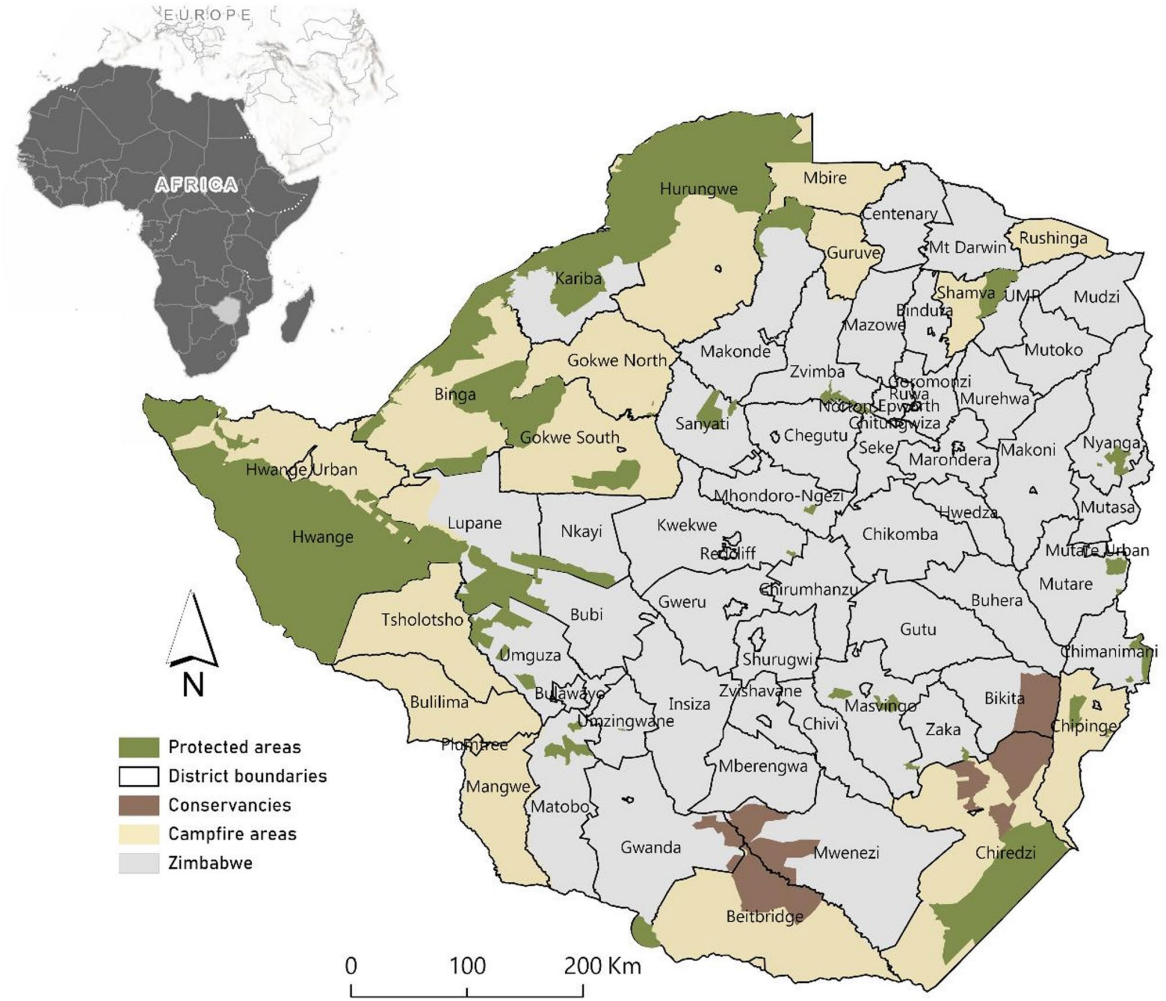
Study area map showing districts and various wildlife sources including protected areas, conservancies and Campfire areas in Zimbabwe. Map was designed by the first author using ArcGIS Pro 3.2.2 (www.pro.arcgis.com).

The study was conducted in Zimbabwe, which is located in southern Africa. The study area spans approximately 390,757 square kilometres and is bordered by Zambia, Mozambique, South Africa, and Botswana^53^ (Fig. 1). The country’s diverse landscapes include savannahs, woodlands, and mountainous regions, with a climate that ranges from temperate in the central plateau to hot in the low-lying areas.

Zimbabwe’s biodiversity is mainly concentrated in protected areas, such as Hwange, Mana Pools, Matusadonha, Chizarira, Zambezi and Gonarezhou national parks, and Hurungwe, Charara, Chirisa, Chete, Matetsi safari areas, and Kariba Recreational Park. These protected areas support several megafaunal species including the African elephant, hereafter elephant, lion, spotted hyena (*Crocuta crocuta*), hippopotamus, hereafter hippo, and Nile crocodile hereafter crocodile. These species are frequently involved in HWC among communities living near wildlife habitats or protected areas^5^.

### Data collection

To understand human and wildlife interactions resulting in HWC, the study utilised the comprehensive HWC databases, which comprise nationwide HWC reports within and outside protected areas consolidated from responsible authorities, including rural district councils and the police. This data was made available by the Zimbabwe Parks and Wildlife Management Authority (ZimParks) for the period covering January 2016 to December 2022. This dataset comprises verified records of HWC incidents, aggregated at the district level on an annual basis. Each record includes details on the species involved, district location, number of people injured or killed (fatalities), and instances of livestock loss. The data is compiled through a structured, cascading reporting system that draws from multiple sources, including communal areas, local authorities, law enforcement agencies, and public health centres. For example, in the event of a fatality in a communal area, the village head reports the incident to the local councillor, who is affiliated with the Rural District Council (RDC). The RDC, in turn, reports such cases along with other HWC-related incidents to ZimParks. Given this coordinated and multi-tiered reporting structure, we consider the dataset to be generally reliable for understanding patterns of HWC in Zimbabwe. To ensure analytical rigor, we included only those records that contained sufficient spatio-temporal detail, specifically, the year and location of each incident, necessary for meaningful analysis. As a result, our study focused on 322 confirmed records of human fatalities involving six megafaunal species: crocodile, elephant, buffalo, lion, hippo, and hyena. This focus reflects both the severity and frequency of fatal encounters with these species, as documented in previous studies^22,30,53^. We acknowledge, however, that the exclusion of incomplete or inconsistent records may have resulted in the underrepresentation of certain species and regions.

### Data analysis

#### Chi-square test

To test the contribution of each species to human fatalities, we calculated the total number of times each species was involved in human fatalities during the study period. The proportional contribution of each species was then assessed as a percentage of the total number of human fatalities due to megafaunal species. The chi-squared test was used to evaluate the statistical significance of the contribution of each species. The test was chosen based on its wide use in evaluating proportional contributions in previous HWC studies^54,55^ and its appropriateness for categorical data which helps to determine whether the observed distribution of fatalities among species deviates from what would be expected by chance. The test was conducted using the following equation:

1 $$\chi^{2} = \sum \frac{{\left( {Oi - Ei} \right)2}}{Ei}$$

where O_i_ represents the observed frequency of fatalities for each species, and E_i_ represents the expected frequency under the null hypothesis of equal contribution by all species.

#### Mann–Kendall test

To analyze temporal trends in human fatalities due to HWC, we used the Mann–Kendall test based on the number of fatalities per year. This non-parametric test is useful for identifying trends in time series data without assuming any specific distribution, making it suitable for our data, which may not follow a normal distribution^56,57^. The Mann–Kendall test has been widely used to detect trends in numerous HWC studies^58,59^. This study used the Mann–Kendall test to determine whether there was a statistically significant increase or decrease in human fatalities over the study period, both for individual species and all species combined. The test was conducted using the following equation:

2 $$S = \mathop \sum \limits_{k = 1}^{n - 1} \mathop \sum \limits_{ j = k + 1}^{n} sgn\left( {x_{j} - x_{x} } \right)$$

where sgn (xj − xk) represents the sign function that returns 1, 0 or − 1 based on the difference between xj and xk. The test produces a test statistic (S), the corresponding *p*-value, and Sen’s slope estimator (Q) to quantify the magnitude of the trend. The Sen’s slope estimator provides the rate of change, with a positive value indicating an increasing trend in fatalities over time and a negative value indicating a decreasing trend. A significant *p*-value (*p* < 0.05) indicates a statistically significant trend in the number of fatalities over time.

#### Getis-Ord Gi* statistics

To identify the hotspots of HWC incidents based on the number of events per district, the study employed the Getis-Ord Gi* statistic. This spatial analytical method is effective for detecting statistically significant clusters of high or low HWC events, which is essential for informing targeted mitigation strategies^60–63^. Its application extends beyond HWC mapping, having been successfully used to identify hotspots of wildlife road mortality^64^ and wildlife disease outbreaks^65^, as well as in recent analyses of HWC spatial patterns^66^. By identifying significant hotspots of fatalities using hotspot analysis, we can better understand the spatial dynamics of HWC and allocate resources more strategically. The Getis-Ord Gi* was conducted using the following equation:

$$Gi* = \frac{{\sum _{{j = 1}}^{n} w_{{ij}} x_{j} - X\sum _{{j = 1}}^{n} w_{{ij}} }}{{S\sqrt {\frac{{[n\sum _{{j = 1}}^{n} w_{{ij}}^{2} - \left( {\sum _{{j = 1}}^{n} w_{{ij}}^{2} } \right)^{2} }}{{n - 1}}} }}$$

where x_j_ is the attribute value (fatalities) at location j, W_ij_ is the spatial weight between locations i and j. X is the mean of the attribute values and S is the standard deviation of the attribute values.

The analysis produces Getis-Ord Gi* statistic (G*) and associated z-scores and *p*-values. A positive z-score (typically greater than 1.96 for a significance level of 0.05) indicates significant clustering of high values (hotspots) in a given area, while a negative z-score indicates clustering of low values (cold spots). A *p*-value less than 0.05 supports the identification of statistically significant clustering patterns.

## Results

### Proportional contributions to fatalities

**Fig. 2:**
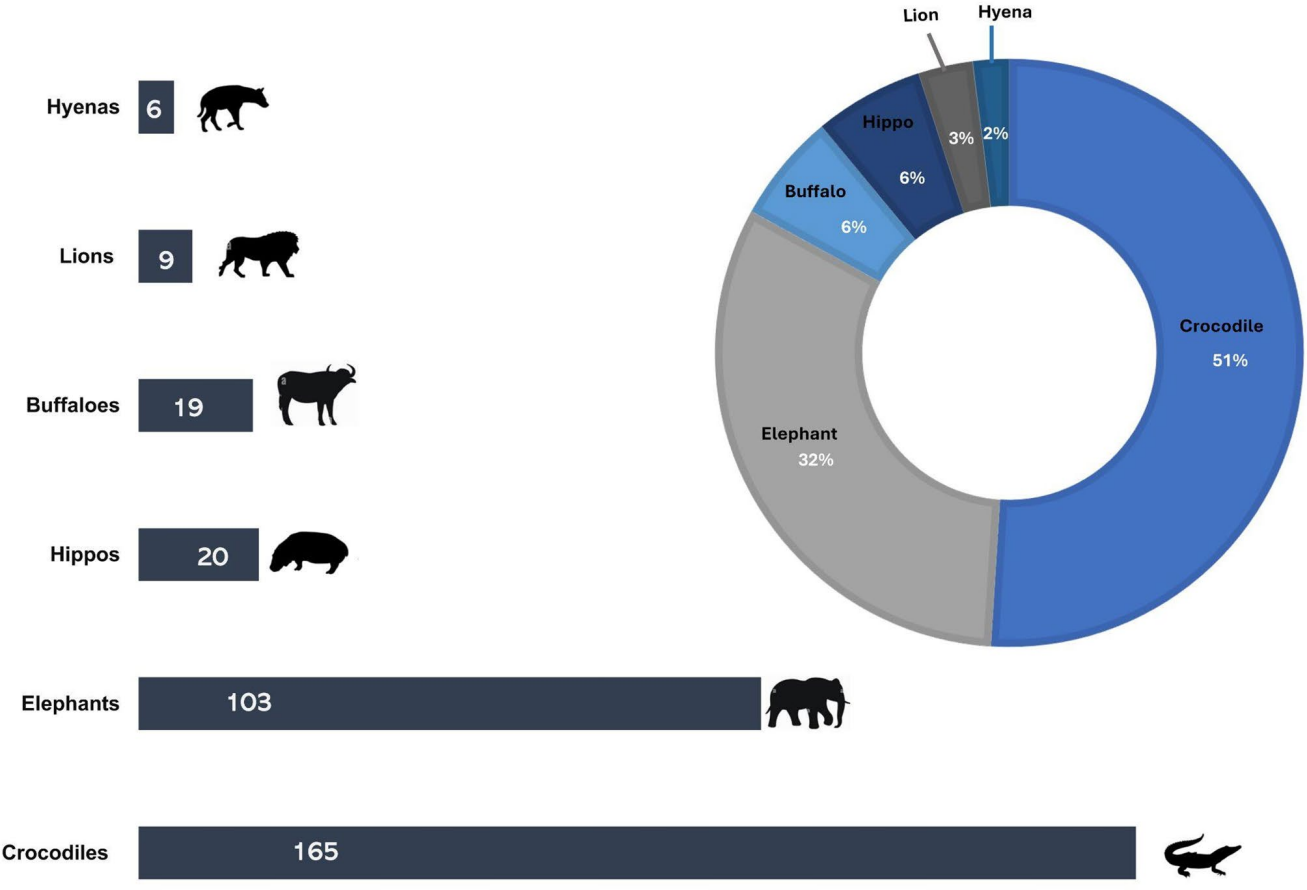
Contribution of megafaunal wildlife species to human fatalities.

The results showed a significant disparity in the proportional contributions of different megafaunal species to total human fatalities (*p* < 0.05). Crocodiles and elephants emerged as the leading contributors, responsible for approximately 83% of the fatalities, with crocodiles accounting for about 51% and elephants for 32%. In contrast, other species, such as lions (3%) and spotted hyenas (2%) were found to contribute significantly less to the overall fatalities (Fig. 2). The chi-squared test confirmed that the observed distribution of fatalities among species is statistically significant (*p* < 0.05), indicating that crocodiles and elephants are significantly involved in fatal encounters with humans compared to other species.

### Temporal trends in fatalities

**Fig. 3:**
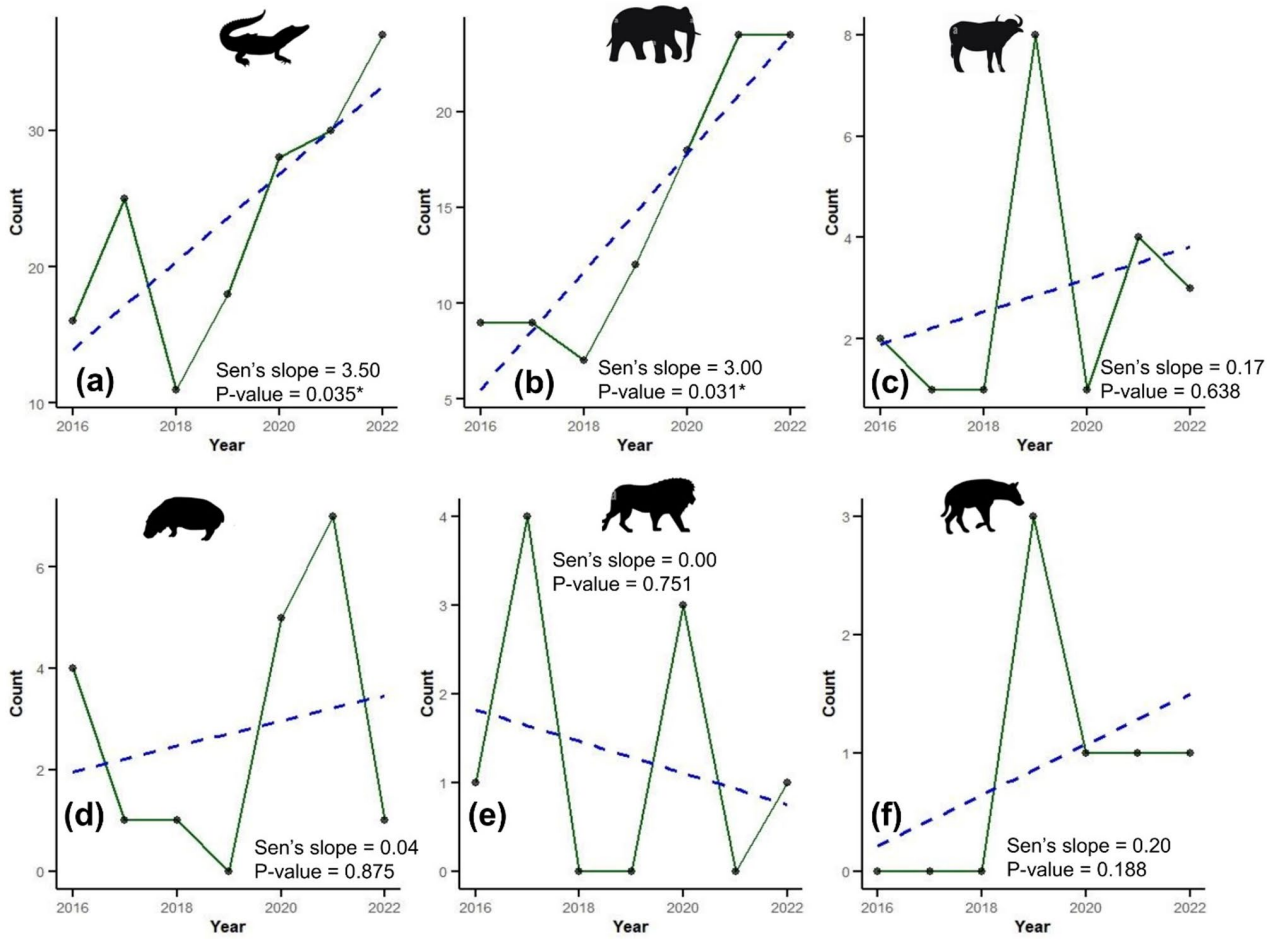
Trends in HWC related human fatalities for the period between 2016 and 2022 for (**a**) crocodile, (**b**) elephant, (**c**) buffalo, (**d**) hippo, (**e**) lion, (**f**) hyena. The green line shows the plot based on data values, and the blue dashed line shows the trend line.

The temporal analysis using the Mann–Kendall test indicated a statistically significant increasing trend in fatalities involving crocodiles and elephants over the study period (*p* < 0.05) (see Fig. 3). For crocodiles, the Sen’s slope estimate was 3.5, indicating an upward trend from 16 fatalities in 2016 to 37 in 2022 (Fig. 3). Similarly, fatalities due to elephants increased from 9 in 2016 to 24 in 2022, with Sen’s slope estimate of 3.0 (Fig. 3). Conversely, other species such as hippos, buffaloes, lions, and hyenas showed insignificant changes in fatality rates over the same period (*p* > 0.05).

**Fig. 4:**
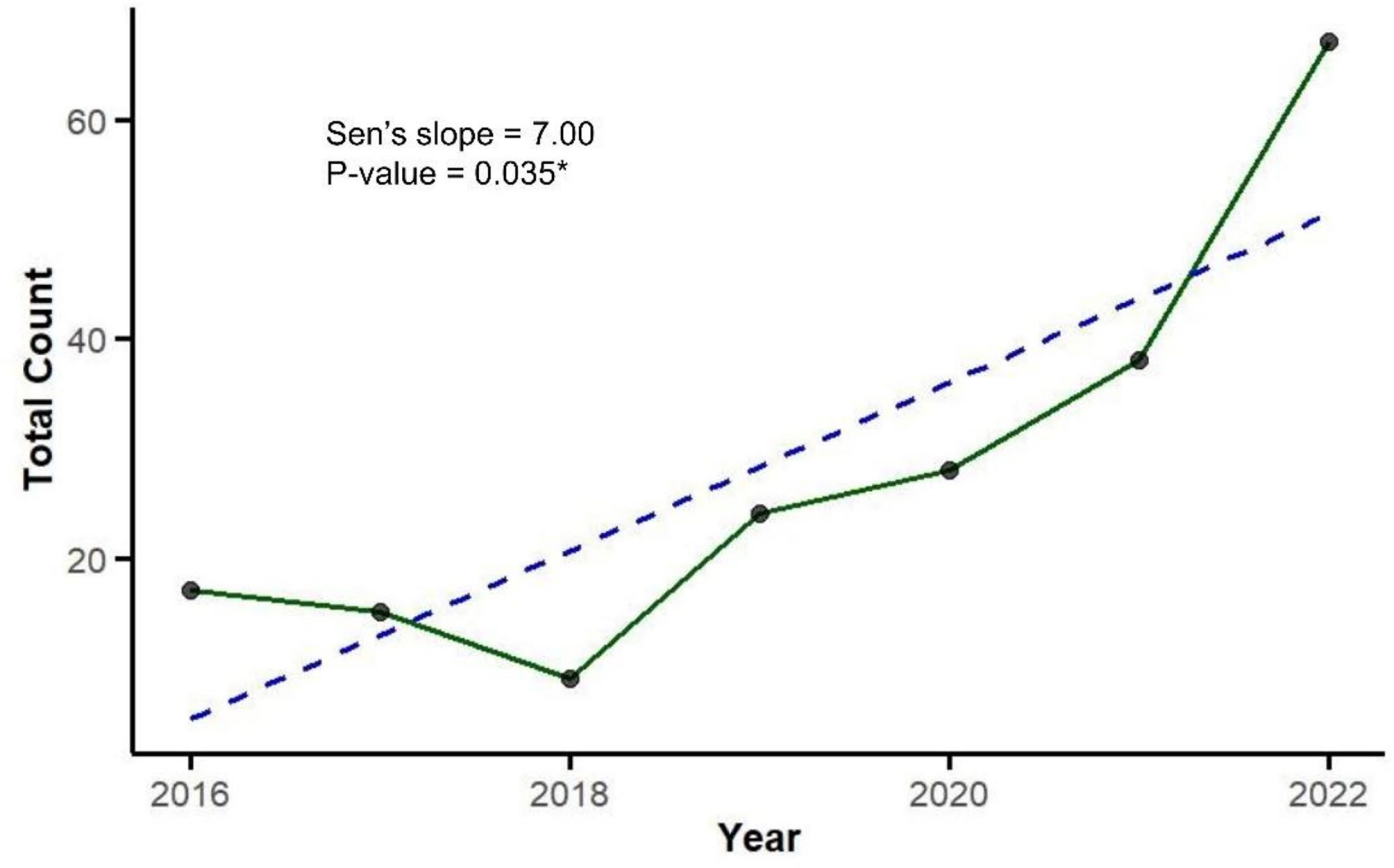
Trends in the total HWC related human fatalities for the period between 2016 and 2022.

Overall, the data reveal a significant upward trend in all HWC-related fatalities across all species, with total fatalities increasing from 17 to 67 per year (Sen’s slope = 7.0, *p* < 0.05) (See Fig. 4). This trend suggests that without intervention, HWC may escalate, further endangering both human lives and wildlife populations.

### Geographical distribution of fatalities

**Fig. 5:**
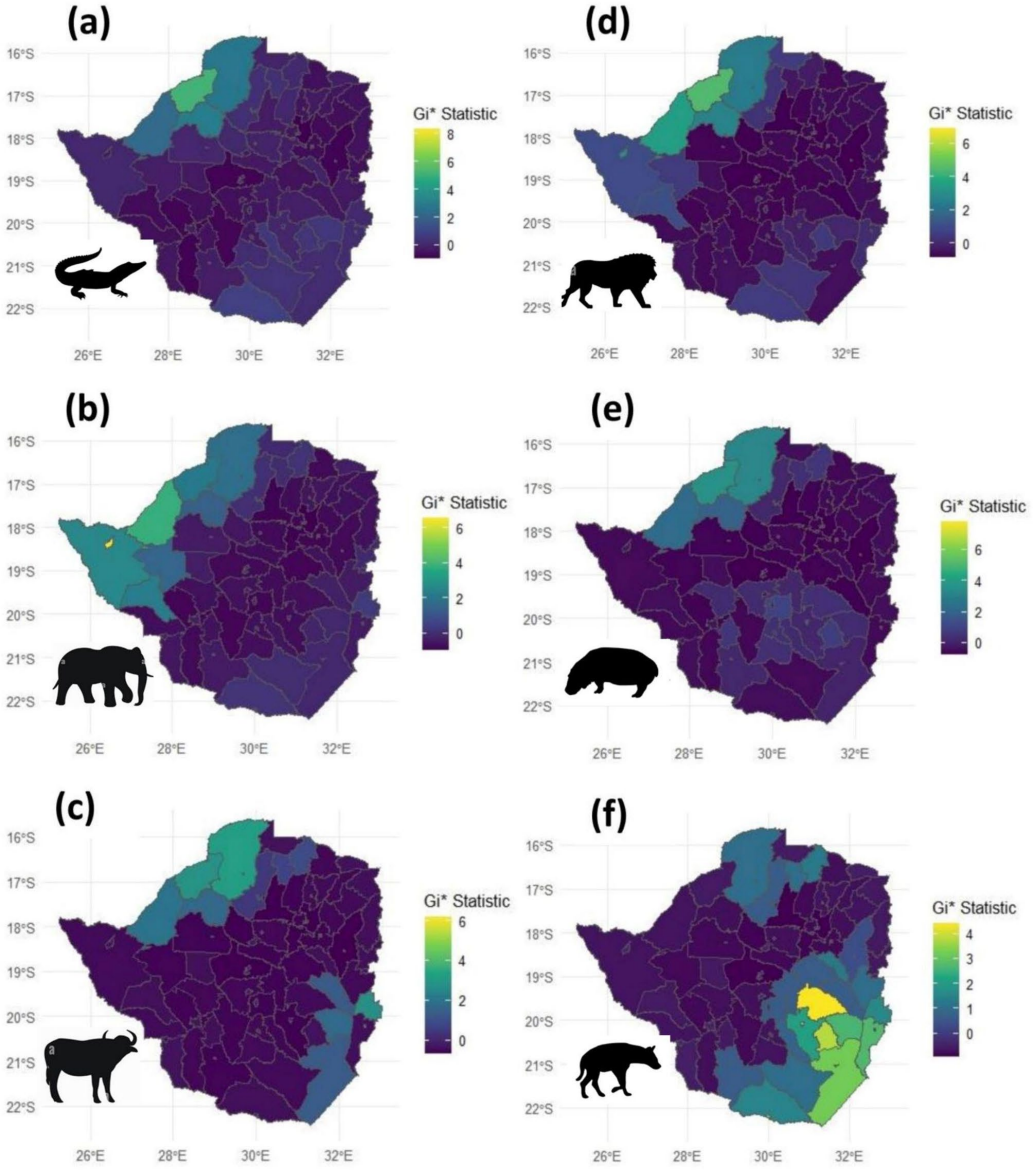
Spatial distribution of hotspots and cold spots for human fatalities across districts of Zimbabwe due to megafaunal species, namely: (**a**) crocodile, (**b**) elephant, (**c**) buffalo, (**d**) lion, (**e**) hippo, (**f**) hyena. The Gi* statistic is a z-score which indicates the intensity of direction of spatial clustering, where high Gi* values (warm colours) show significant hotspots and low values (cold colours) show significant cold spots. Maps were designed by the first author using R-studio 2024.09.0 (www.posit.co/rstudio-desktop).

Results for the Getis-Ord Gi* statistics identified significant hotspots and coldspots for fatalities due to crocodile, elephant, buffalo, lion, hippo and hyena (Fig. 5). Significant hotspots for fatalities due to crocodile, elephant, buffalo, lion and hippo were situated in both the northern and western districts of Zimbabwe, namely Hurungwe, Kariba, Binga, Hwange and Gokwe (North and South) districts. For fatalities due to buffalo and hyena, significant hotpots were observed in the south-eastern districts of Zimbabwe namely Chiredzi, Bikita, Chipinge, Buhera and Beitbridge. All the species had a hotspot for fatalities in the northern districts of Zimbabwe namely Hurungwe and Kariba. On the other hand, coldspots for fatalities were consistently observed in areas stretching from the northeastern districts (~ Centenary, Guruve and Rushinga) through the central districts (~ Kwekwe, Chegutu and Gweru) down to south western districts (~ Mangwe, Matobo, Gwanda) of the country.

**Fig. 6:**
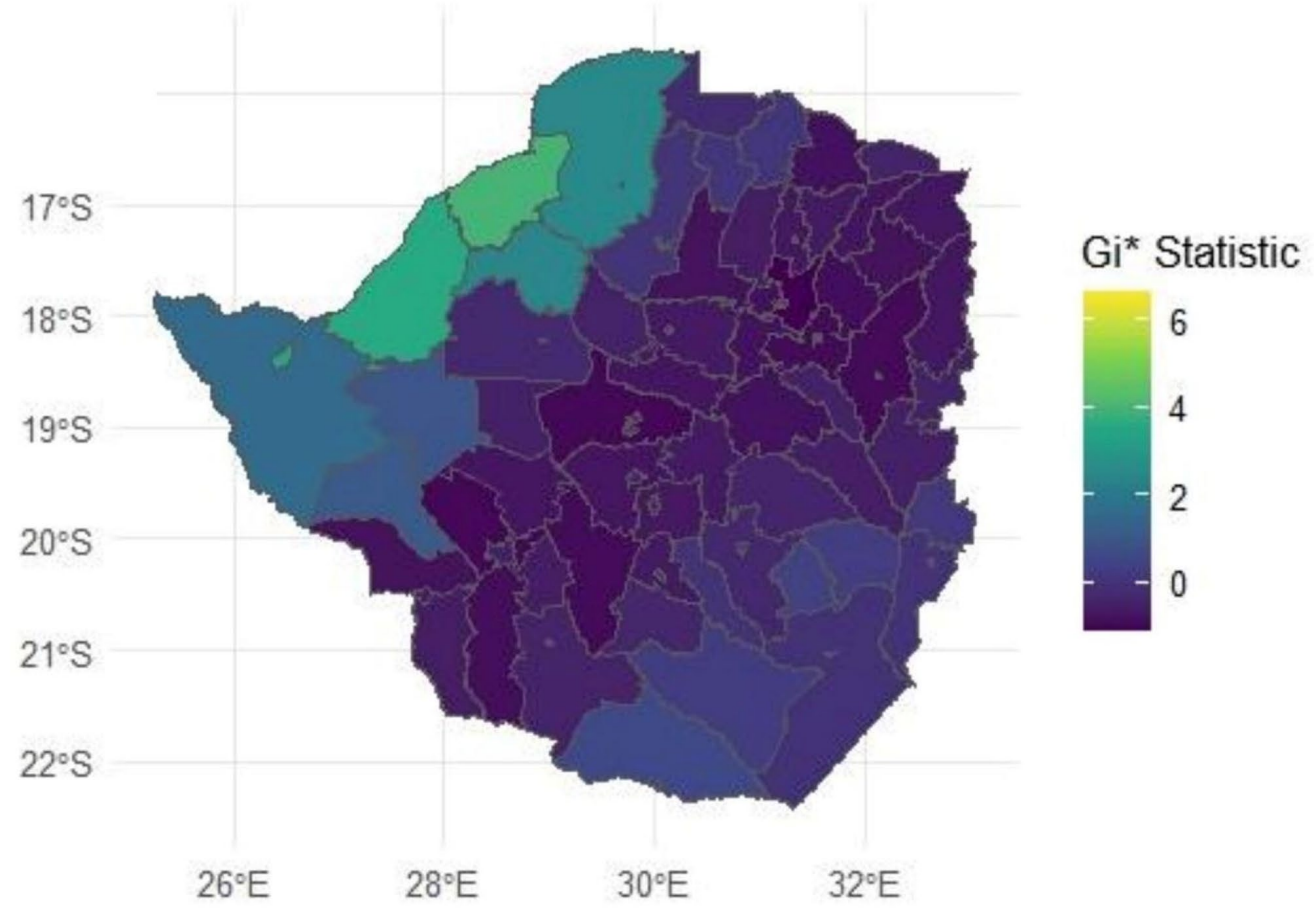
The spatial distribution of hotspots and cold spots of human fatalities from HWC across districts of Zimbabwe. The Gi* statistic is a z-score which indicates the intensity of direction of spatial clustering, where high Gi* values (warm colours) show significant hotspots and low values (cold colours) show significant cold spots. Map was designed by the first author using R-studio 2024.09.0 (www.posit.co/rstudio-desktop).

Overall, districts located in the northern and western districts of Zimbabwe exhibited significant hotspots for HWC fatalities, particularly involving the five megafaunal species under investigation (See Fig. 6). This suggests a concentrated risk area that may require focused intervention strategies. Notably, these districts have a high concentration of protected areas. In contrast, districts in the northeastern, central, and southwestern regions show significant cold spots of HWC fatalities. These regions are characterized by fewer concentrations of protected areas.

## Discussion

This study offers a comprehensive overview of the role megafaunal species play in human fatalities in Zimbabwe. Our findings reveal that elephants and crocodiles are responsible for over 80% of these fatalities, highlighting the urgent need to reassess and refine current mitigation strategies in areas with elephants and crocodiles. These include implementing more effective deterrent measures, such as electric fences with increased voltage or acoustic deterrents and improving HWC management programs to educate local communities about safe practices and coexistence with these large animals^67^. Additionally, investing in habitat restoration and maintaining wildlife corridors can help reduce human–wildlife encounters and mitigate the risk of fatalities^68^. While the involvement of these species in crop raiding and livestock depredation is well documented^68–71^, our findings also indicate that these species impact human lives. Understanding human fatalities from HWC is crucial, as this often leads to deep-seated anger, resentment, and retaliatory killings within affected communities^41^. These retaliatory actions not only threaten the survival of the species involved but also undermine broader conservation efforts^72^. For species such as crocodiles and elephants, which already face significant conservation challenges, the escalation of HWC into retaliatory killings can have dire consequences^72–74^. While often abundant in certain regions, crocodiles are still vulnerable to overexploitation and habitat loss^75^. Retaliatory killings can exacerbate these threats, particularly in areas where crocodile populations are already under pressure from human activities such as fishing and habitat encroachment^76^. Elephants are listed as vulnerable by the International Union for Conservation of Nature (IUCN) due to threats from habitat fragmentation, poaching, and human-elephant conflict^77^. Zimbabwe’s elephant population, though large, is not exempt from these pressures^78,79^. Retaliatory killings, driven by the loss of human lives, can contribute to population declines and disrupt social structures within elephant herds, further endangering the species. Therefore, it is imperative to develop and implement targeted interventions that prioritise human safety and wellbeing while safeguarding these key species’ survival. By fostering coexistence, the results from this study can help ensure the long-term conservation of these species and the conservation of the ecosystems they inhabit.

### Temporal distribution

**Fig. 7:**
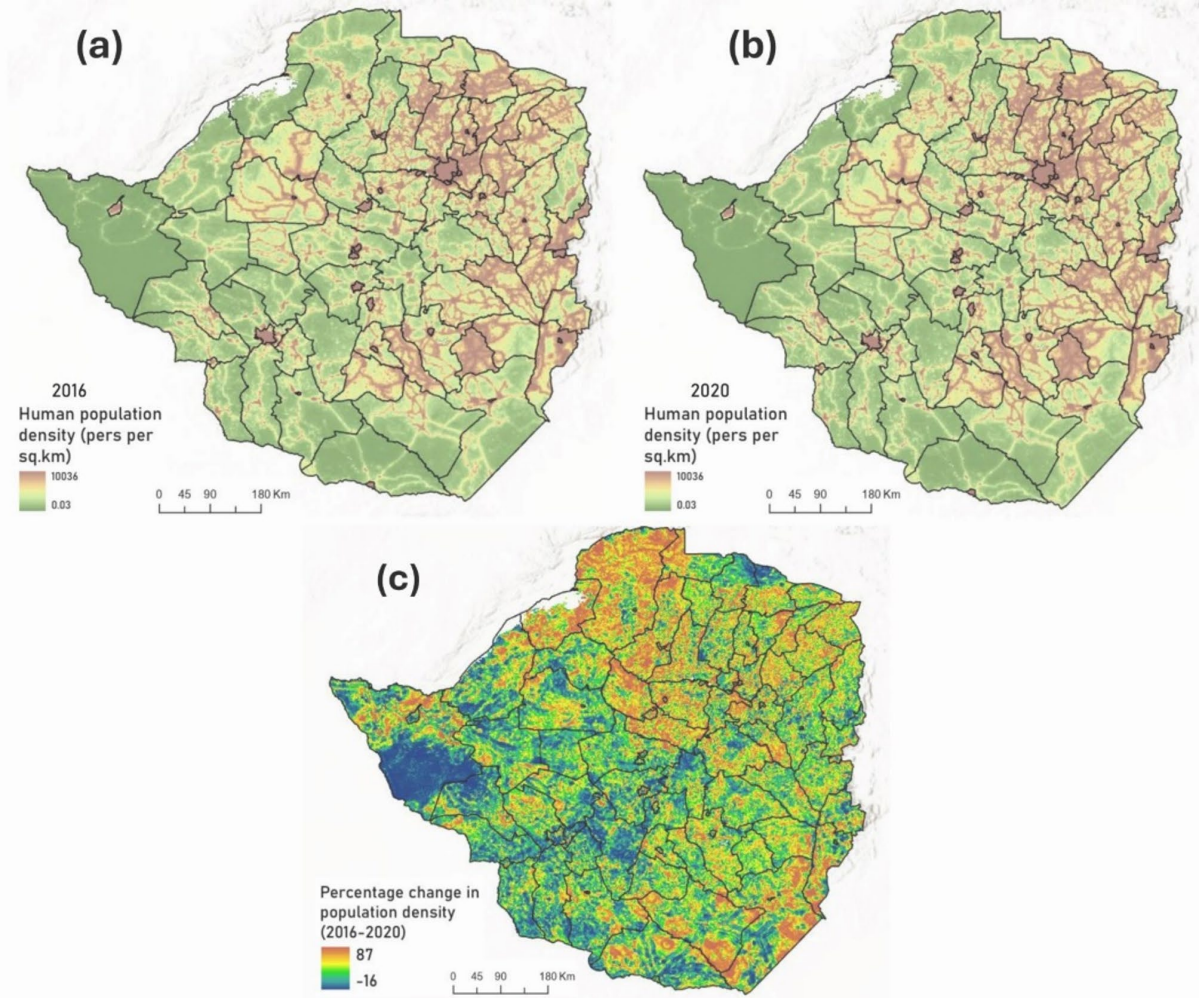
The human population density distribution for Zimbabwe in the years 2016 (**a**) and 2020 (**b**), together with the percentage change in population density during the same period between 2016 and 2020 (**c**). Maps were designed by the first author using ArcGIS Pro 3.2.2 (www.pro.arcgis.com).

Our findings reveal a significant upward trend in fatalities involving crocodiles and elephants over the study period (*p* < 0.05). Many water bodies (e.g., Lake Kariba and Ruti Dam) in Zimbabwe are home to crocodiles and are situated near communities that rely on these waters for fishing, bathing, washing, and collecting water^50,68,78^. Community utilisation of these water resources with inadequate safety measures poses a significant risk of human-crocodile encounters and fatalities^80^. In addition, increasing human population density in this part of the country (See Fig. 7c) could be driving overfishing by local communities. This can decimate fish stocks and drive crocodiles to alternative food sources, particularly increasing the likelihood of fatal encounters^81^. This is also supported by an observation from a study by Matanzima^73^, who showed that most crocodile-related fatalities in Kariba district were in the dry season when crocodiles’ natural habitat and prey base was diminished. This underscores the need for sustainable resource management that considers the rising human population to ensure that fish stocks remain sufficient to support crocodile populations^82^.

The rising fatalities involving elephants could be related to Zimbabwe’s growing elephant population, now exceeding 83,000, the second largest globally after Botswana^83^. As elephant populations grow, so does the demand for space, food, and water, often bringing elephants closer to human settlements^83,84^. This increased interaction increases the risk of conflict, particularly in areas where human activities encroach on traditional elephant ranges or movement corridors^19,85^. These fatalities often occur when humans attempt to protect their crops from elephants^86–88^. These fatal encounters also occur during the dry season, when water scarcity drives elephants closer to human settlements in search of water^89^.

We found no significant trend in human fatalities due to hippo, buffalo, lion and hyenas (*p* > 0.05). This could be because these species typically do not attack humans unless provoked or threatened. Hippos and buffaloes, for example, are generally known to be aggressive primarily when they feel cornered or perceive a threat to their territory or young^89,90^. Additionally, the hippo and buffalo population has significantly declined in the past 10 years especially in the northern parts of the country (see Table S1), thereby limiting chances of their encounters with humans. Lions and hyenas are more likely to target livestock or wild prey rather than humans, and attacks on people often occur when humans unknowingly intrude into their habitat^91,92^. However, some studies have documented increased livestock depredation and non-fatal human encounters with these species^93–95^. Human fatalities, unlike mere attacks or livestock raids, often involve unavoidable, high-risk situations with profound socio-economic and psychological impacts on communities. Therefore, trends in human fatalities warrant particular attention, necessitating targeted conservation and conflict mitigation efforts that prioritize human safety and wellbeing while addressing broader HWC dynamics^96^.

### Spatial distribution

The study observed hotspots for five megafaunal species (crocodiles, elephants, buffaloes, lions, and hippos) in Zimbabwe’s northern and western regions (Fig. 5). The spatial distribution patterns of these hotspots are likely driven by a combination of connectivity, ecological, environmental and anthropogenic factors^96–98^. The northern and western regions are characterised by extensive river systems, such as the Zambezi River, which supplies Lake Kariba providing ideal habitats for crocodiles and hippos, including large herbivores like elephants and buffaloes^100^. These environments support a high density of wildlife and together with a growing human population density which increase the likelihood of human–wildlife interactions, particularly where human activities, such as agriculture and fishing, bring people into proximity to dangerous animals^49^. Data from the world population hub indicates that the human population density in most of the northern and western parts of the country increased by over 50% (See Fig. 7c) during the period between 2016 (Fig. 7a) and 2020 (Fig. 7b). This certainly increased chances of human–wildlife encounters during this same period which recorded a steep rise in HWC-related fatalities (refer to Fig. 4).

Moreover, these regions are closely connected to the proximity of multiple protected areas within the Kavango-Zambezi Transfrontier Conservation Area (KAZA TFCA). KAZA TFCA is the largest transboundary conservation area in the world, spanning five countries: Angola, Botswana, Namibia, Zambia, and Zimbabwe. This region encompasses numerous national parks and wildlife reserves that serve as critical habitats for lions, elephants, and other megafaunal species^101^. The KAZA TFCA facilitates wildlife movement across borders through its vast, interconnected network of protected areas, essential for conserving wide-ranging species such as elephants and lions^102^. However, this connectivity also increases human–wildlife interactions in adjacent human settlements, as elephants and lions often move into areas where human activities have encroached upon traditional wildlife corridors^102,103^. This overlap can lead to more frequent and sometimes fatal encounters between humans and wildlife, particularly in regions such as western Zimbabwe, where the boundaries between protected and communal lands are not always clearly defined^69,104,105^. Improving wildlife corridors in the KAZA TFCA, strengthening of CBNRM programs and enhancing early warning systems to alert communities of nearby wildlife movements could help reduce HWC fatalities and foster coexistence between humans and wildlife in this biologically rich but conflict-prone region.

Southeastern Zimbabwe exhibited fewer HWC fatalities involving most species (crocodiles, elephants, buffaloes, lions, and hippos), except for hyenas, which had their hotspots in this area. This variation could be due to different ecological conditions and human settlement patterns that reduce the frequency of encounters with species such as crocodiles, elephants, buffaloes, lions, and hippos^106–110^. Hyenas, however, are highly adaptable and opportunistic predators that often thrive in areas with high human density due to the availability of anthropogenic food resources^111^. Their ability to scavenge and exploit human waste may explain their hotspots in this region, where they conflict with humans over resources.

The variation in hotspots underscores the need for targeted intervention strategies that address the specific dynamics of HWC in different regions of Zimbabwe. The adaptation of these spatial distribution patterns in formulating strategies for providing psychosocial support to vicarious victims of HWC is of major value. The psychological impact of HWC-related human fatalities is an overlooked dimension of conflict mitigation. The trauma experienced by affected families and communities can drive retaliatory actions, exacerbating the conflict. Therefore, it is imperative that counselling and rehabilitation resources and services be deployed to the northern and western hotspots of Zimbabwe. Furthermore, in hotspot areas, efforts could focus on mitigating risks associated with water-based activities and reducing conflicts with large herbivores and predators through community-based management and early warning systems^111–113^. Conversely, in the southeastern region, interventions could prioritize waste management and securing livestock to reduce hyena-related conflicts. Tailoring strategies to the unique ecological and social contexts of each region will be crucial for effectively reducing HWC fatalities, restoring a sense of wildlife ownership in communities and promoting coexistence between humans and wildlife^92^. These findings have broader implications for HWC management across Africa, where megafaunal species frequently come into contact with human populations. By developing data-driven, species-specific strategies, other countries facing similar conflicts can benefit from the approaches suggested in this study, ultimately reducing retaliatory killings and enhancing conservation outcomes.

### Limitations and recommendations

While this study provides valuable insights into the spatio-temporal dynamics of megafaunal species in Zimbabwe, it lacks an important temporal dimension: the consideration of seasonal variation. Understanding seasonality is crucial for planning and implementing targeted interventions. For example, crocodile attacks often peak during the dry season when water levels are lower, and the water becomes murky and muddy, increasing the likelihood of human encounters with these animals^114^. Also, elephants and buffaloes are known to exhibit increased aggression during the dry season due to intensified competition for limited water and food resources, potentially skewing conflict data towards certain periods^115^. Similarly, the movement patterns of lions and hyenas during the wet season when their natural prey may migrate or become less accessible can result in increased proximity to human settlements, leading to higher instances of livestock depredation^116^. These seasonal dynamics were not explicitly accounted for in the analysis and may affect the interpretation of spatial and temporal HWC patterns.

We could not evaluate the effect of seasonality of these species in our study due to missing records on the place, month and day of each attack. This lack of detailed spatial–temporal data limits our ability to identify patterns that could be critical for mitigating human–wildlife conflict. In the process of cleaning the dataset for analysis, we may have inadvertently excluded relevant records from key regions due to missing or inconsistent information. This may have resulted in underrepresentation of certain areas, particularly the southeastern regions of Zimbabwe, which support significant wildlife populations. Notable among these are communities surrounding Gonarezhou National Park, Save Valley Conservancy, Bubye Valley Conservancy, and Nuanetsi Ranch. The absence of comprehensive data from these areas may have influenced the spatial distribution patterns observed in our analysis. Future studies should include precise spatial–temporal information, such as the specific dates and coordinates of incidents to accurately assess seasonal trends and patterns in human–wildlife conflicts. To address loss of data along bureaucratic protocols followed when reporting HWC-related fatalities, we recommend the development of a national HWC monitoring and reporting system which ensures more centralised, standardized and consistent recording of data. Reliable data would facilitate crafting of more timely and effective interventions. For instance, implementing seasonal warnings, increasing patrolling during high-risk periods, or temporarily restricting high-risk activities like fishing or farming near water bodies during peak conflict times could significantly reduce human fatalities^93^.

## Conclusion

This study concludes that elephants and crocodiles are the leading causes of human fatalities in Zimbabwe, accounting for over 80% of such incidents. By establishing a national baseline for these critical issues, this research paves the way for more effective, targeted interventions to improve human–wildlife coexistence. Specifically, maps for fatality hotspots should guide deployment of psychosocial support services for vicarious victims of HWC. Future strategies should leverage detailed temporal data and address ecological factors to enhance conflict resolution efforts.

## Electronic supplementary material

Below is the link to the electronic supplementary material.

Supplementary Material 1

## Data Availability

The data presented in this study is available on request from the corresponding author.
