# Supplementary material for: Mapping human fatalities from megafauna to inform coexistence strategies
Source: Sci Rep. 2025 Sep 30;15:33856. doi: 10.1038/s41598-025-04934-0 (PMC12485064; doi:10.1038/s41598-025-04934-0)
Supplement: Supplementary file 1 — Supplementary Material 1 [file 41598_2025_4934_MOESM1_ESM.pdf]

Supplementary material

Table S1: List of population trends for the studied species based on records from the Zimbabwe Parks and Wildlife database.

Species	Population trend (2016–2025)	Notes
Elephant	Increasing	Population grew from around ~82,092 (2014) to over ~84,000 (2023)
Buffalo	Decreasing	Decline from ~50,000 to 30,000–35,000- northern populations affected more.
Lion	Uncertain	Data limited-conservation efforts are ongoing in protected areas.
Hippo	Decreasing	Significant decline along the Zambezi River- from ~6,000 (2002) to 1,424 (2022).
Crocodile	Unknown	Lack of recent comprehensive data.
Hyena	Unknown	Limited data on population trends.

Table S2: Detailed list of HWC-related fatalities per district in Zimbabwe, that were used in mapping the spatial hotspots for mortalities the 6 species: elephant, buffalo, lion, hippo, crocodile and hyena.

District names	elephant	crocodile	buffalo	hippo	hyena	lion
Beitbridge	6	5	0	0	2	1
Bikita	0	0	0	0	1	0
Bindura	0	1	0	0	0	0
Binga	1	16	0	0	0	0
Bubi	0	0	0	0	0	0
Buhera	0	2	0	0	1	0
Bulawayo	0	0	0	0	0	0
Bulilima	0	0	0	0	0	0
Centenary	0	0	0	0	0	0
Chegutu	0	2	0	0	0	0
Chikomba	0	1	0	0	0	0
Chimanimani	0	0	0	0	0	0
Chinhoyi	0	2	0	0	0	0
Chipinge	4	4	2	0	0	0
Chiredzi	9	18	4	4	0	1
Chirumhanzu	0	0	0	0	0	0
Chitungwiza	0	0	0	0	0	0
Chivi	0	2	0	0	0	0
Epworth	0	0	0	0	0	0
GokweNorth	0	0	0	0	0	0

GokweSouth	1	0	0	0	0	0
GokweSouthUrban	1	0	0	0	0	0
Goromonzi	0	0	0	0	0	0
Guruve	0	1	0	0	0	0
Gutu	0	1	0	0	0	0
Gwanda	0	0	0	0	0	0
Gweru	0	0	0	0	0	0
HarareUrban	0	0	0	0	0	0
Hurungwe	7	8	0	3	0	2
Hwange	33	0	0	0	0	2
Hwedza	0	0	0	0	0	0
Insiza	0	0	0	2	0	0
Kariba	28	51	7	6	0	2
Kwekwe	1	2	0	0	0	0
Lupane	0	0	0	0	0	0
Makonde	1	3	0	0	0	0
Makoni	0	1	0	0	0	0
Mangwe	0	0	0	0	0	0
Marondera	0	0	0	0	0	0
Masvingo	0	10	0	2	1	0
MasvingoUrban	0	1	0	0	0	0
Matobo	0	2	0	0	0	0
Mazowe	0	0	0	0	0	0
Mberengwa	0	0	0	0	0	0
Mbire	7	9	6	3	1	1
Mhondoro-Ngezi	0	0	0	0	0	0
MountDarwin	1	2	0	0	0	0
Mudzi	0	0	0	0	0	0
Murehwa	0	0	0	0	0	0
Mutare	2	0	0	0	0	0
Mutasa	0	0	0	0	0	0
Mutoko	0	0	0	0	0	0
Mwenezi	0	7	0	0	0	0
Nkayi	0	0	0	0	0	0
Norton	0	0	0	0	0	0
Nyanga	0	0	0	0	0	0
Plumtree	0	0	0	0	0	0
Redcliff	0	0	0	0	0	0
Rushinga	0	1	0	0	0	0
Ruwa	0	0	0	0	0	0
Sanyati	0	0	0	0	0	0
Seke	0	0	0	0	0	0
Shamva	0	0	0	0	0	0
Shurugwi	0	0	0	0	0	0
Tsholotsho	0	0	0	0	0	0

Umguza	0	0	0	0	0	0
Umzingwane	0	0	0	0	0	0
UzumbaMarambaPfungwe	0	0	0	0	0	0
Victoria Falls	0	0	0	0	0	0
Zaka	0	0	0	0	0	0
Zvimba	0	12	0	0	0	0
Zvishavane	0	1	0	0	0	0
**Total sum**	**103**	**165**	**19**	**20**	**6**	**9**
